# A Two-Step Feature Selection Radiomic Approach to Predict Molecular Outcomes in Breast Cancer

**DOI:** 10.3390/s23031552

**Published:** 2023-01-31

**Authors:** Valentina Brancato, Nadia Brancati, Giusy Esposito, Massimo La Rosa, Carlo Cavaliere, Ciro Allarà, Valeria Romeo, Giuseppe De Pietro, Marco Salvatore, Marco Aiello, Mara Sangiovanni

**Affiliations:** 1 IRCCS SYNLAB SDN, Istituto di Ricerca Diagnostica e Nucleare, Via E. Gianturco 113, 80143 Naples, Italy; 2 Institute for High Performance Computing and Networking, National Research Council of Italy (ICAR-CNR), Via P. Castellino 111, 80131 Naples, Italy; 3 Bio Check Up S.r.l., Via Riviera di Chiaia 9a, 80122 Naples, Italy; 4 Department of Advanced Biomedical Sciences, University of Naples Federico II, 80131 Naples, Italy

**Keywords:** radiomics, Breast Cancer, machine learning, feature selection

## Abstract

Breast Cancer (BC) is the most common cancer among women worldwide and is characterized by intra- and inter-tumor heterogeneity that strongly contributes towards its poor prognosis. The Estrogen Receptor (ER), Progesterone Receptor (PR), Human Epidermal Growth Factor Receptor 2 (HER2), and Ki67 antigen are the most examined markers depicting BC heterogeneity and have been shown to have a strong impact on BC prognosis. Radiomics can noninvasively predict BC heterogeneity through the quantitative evaluation of medical images, such as Magnetic Resonance Imaging (MRI), which has become increasingly important in the detection and characterization of BC. However, the lack of comprehensive BC datasets in terms of molecular outcomes and MRI modalities, and the absence of a general methodology to build and compare feature selection approaches and predictive models, limit the routine use of radiomics in the BC clinical practice. In this work, a new radiomic approach based on a two-step feature selection process was proposed to build predictors for ER, PR, HER2, and Ki67 markers. An in-house dataset was used, containing 92 multiparametric MRIs of patients with histologically proven BC and all four relevant biomarkers available. Thousands of radiomic features were extracted from post-contrast and subtracted Dynamic Contrast-Enanched (DCE) MRI images, Apparent Diffusion Coefficient (ADC) maps, and T2-weighted (T2) images. The two-step feature selection approach was used to identify significant radiomic features properly and then to build the final prediction models. They showed remarkable results in terms of F1-score for all the biomarkers: 84%, 63%, 90%, and 72% for ER, HER2, Ki67, and PR, respectively. When possible, the models were validated on the TCGA/TCIA Breast Cancer dataset, returning promising results (F1-score = 88% for the ER+/ER− classification task). The developed approach efficiently characterized BC heterogeneity according to the examined molecular biomarkers.

## 1. Introduction

Breast Cancer (BC) is the most commonly diagnosed cancer type in the world. The most recent global cancer statistics estimate that there are about 2.3 million incident BC cases and that the disease is the leading cause of cancer mortality in women worldwide [1]. Currently, radiographic evaluation followed by a histological confirmation of malignancy on biopsy samples is used to make the early diagnosis of BC [2,3]. Although this method allows practitioners to safely and effectively characterize the molecular changes in breast tissue, it has intrinsic drawbacks because of the accessibility and heterogeneity of the tumors and the risks associated with the bioptic process [4].

In particular, it is well-known that the heterogeneity of BC, which also depends on the temporal variation, may lead to the failure of cancer treatments and poor prognoses [5].

The identification of numerous biomarkers through tissue biopsy or medical imaging is necessary for assessing heterogeneity at early diagnosis, with the correct classification of the tumor genotype being of fundamental importance for the clinical management of this pathology [6]. However, differently from imaging, the risks of invasive procedures, focal sampling errors, and tumoral characteristics (such as small size, location, or heterogeneous necrosis) represent relevant drawbacks associated with biopsies. In this perspective, radiomic techniques may notably support the non-invasive management of BC [7].

Radiomics was defined as “the high-throughput extraction of large amounts of image features from radiographic images” [8]. It could be used to analyze both temporal and spatial BC heterogeneities through the quantitative evaluation of the radiologic images. Recent developments in radiomics analysis showed the potential to retrieve useful incremental information from standard imaging data in a non-invasive way.

Radiomics can be successfully applied to both morphological (such as T2-weighted—T2—images) and functional magnetic resonance images (MRI) (such as dynamic contrast-enhanced (DCE) and diffusion-weighted imaging (DWI)) to predict histological outcomes in BC [9,10].

MRI is the reference imaging modality for soft tissue characterization, with functional techniques such as DCE-MRI and DWI greatly supporting the characterization of the anatomic and functional properties of BC [11,12]. In particular, MRI radiomics has been used to predict malignancy, molecular subtypes, complete pathological response to neoadjuvant chemotherapy, and metastasis in BC. However, both diagnostic and prognostic outcomes depend on the underlying biological characteristics of BC.

The accurate examination of BC biology is fundamental since each BC is characterized by a unique biological and genetic profile, thus corresponding to a wide range of prognoses and therapeutic options. Different molecular profiles, proliferative rates, tumor receptors, and grades define subtypes. The Estrogen Receptor (ER), Progesterone Receptor (PR), Human Epidermal Growth Factor Receptor 2 (HER2), and Ki67 antigen are the four biomarkers routinely examined in BC biopsies and excision specimens due to their potential impact on heterogeneity prognosis and clinical therapy. HER2-positive (HER2+) breast cancers are more aggressive and show a poorer prognosis than HER2-negative (HER2−) cancers. Positive hormonal receptor status, such as in ER-positive (ER+) and PR-positive (PR+) tumors, presents lower risk of mortality than ER-negative (ER−) and/or PR-negative (PR−) diseases. Ki67 is a proliferative index of BC, and a high Ki67 level is associated with an elevated relapse rate and worse survival [13].

There is a growing scientific production exploring different radiomic approaches to predict molecular outcomes of BC on MRI datasets [14,15,16,17,18,19,20]. Nevertheless, a common drawback affecting the largest part of these works was related to the absence of comprehensive sets of molecular markers and MRI modalities that would allow for an effective comparison of different models and feature selection approaches [9]. For instance, The Cancer Genome Atlas BReast invasive CArcinoma (TCGA-BRCA) dataset [21], collected by the TCGA/TCIA project, despite its limited size, still represents the largest publicly available set of breast MRI providing also clinical, pathological, and genomic data. However, TCGA-BRCA lacks information on the Ki67 molecular marker and does not include the DWI sequence.

This work aimed to develop a new radiomic approach based on a two-step feature selection process to predict the most routinely examined BC biomarkers (ER, PR, HER2, Ki67) and compare the prediction models’ performances in different settings. It exploited a comprehensive dataset that includes multiparametric MRI (mpMRI) images from morphological T2, functional DCE-MRI images, and Apparent Diffusion Coefficient (ADC) maps from DWI, as well as all four relevant molecular markers for BC management.

## 2. Materials and Methods

### 2.1. Study Design

**Figure 1:**
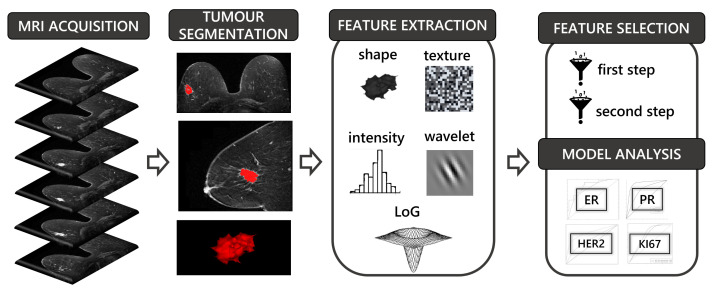
The steps of the adopted radiomics pipeline. They include MRI acquisition, tumor segmentation, feature extraction, feature selection, and model analysis.

The goal of the proposed methodology was to build robust predictors for the four biomarkers commonly used in BC molecular profiling. Two comprehensive datasets were exploited: the MOLIM ONCO BRAIN dataset (DS${}_{M}$) for model development and the TCGA-BRCA (DS${}_{T}$) for model validation. Both included: (i) mpMRI preoperative images of BC patients (both functional and morphological sequences), (ii) clinical information related to at least three biomarkers (ER, PR, HER2), and (iii) BC tumour segmentations. Figure 1 shows the steps of the radiomic pipeline used to carry out the model development and validation: mpMRI images acquisition (Section 2.3), tumor segmentation (Section 2.4), radiomic feature extraction (Section 2.5), a two-step feature selection and classification (Section 2.6), and best model selection and validation using an external dataset (Section 2.7).

### 2.2. Patients

The patient cohort included two datasets of BC patients. For the DS${}_{M}$, 92 MRI pre-operative examinations of patients with BC (93 lesions) were collected from February 2017 to February 2020 and retrospectively evaluated. Inclusion criteria were the following: (1) age >18 years and (2) patients with histologically proven BC. Patients were excluded if the histological report was unavailable and the MRI images were significantly affected by motion artifacts. All patient information was de-identified before the data were stored in the collection of BCU Imaging Biobank [22]. The study was approved by the Ethical Committee IRCCS Pascale (Prot. 12/19 OSS SDN), and written informed consent was obtained from all participants.

The DS${}_{T}$ was used to perform model validation. It included 164 MRI studies (Digiatl Imaging and COmmunications in Medicine—DICOM—format, 88.1 GB) of 139 Breast Cancer patients from several American hospitals and clinics. The clinical, genetic, and pathological data were acquired from the Genomic Data Commons Data Portal [23]. To reduce potential image acquisition variation, only breast MRI studies that were similar in acquisition and technique (namely, MRIs that were acquired on a 1.5 T magnet strength using GE (GE Medical Systems, Milwaukee, WI, USA) scanners and protocols) were analyzed. This selection procedure resulted in a total of 93 patients. For these cases, tumor segmentations were available in binary format [24]. One subject with missing DCE images and one with missing genotyping data were excluded from the study. Finally, the DS${}_{T}$ consisted of 91 BC patients. The images and segmentations were downloaded and converted in NIfTI (Neuroimaging Informatics Technology Initiative) format.

### 2.3. MRI Acquisition

MRI examinations of the DS${}_{M}$ were performed using a 3 T Biograph mMR (Siemens Healthcare, Erlangen, Germany) with a dedicated breast surface coil. T2 Turbo spin-echo (TSE T2) sequence was acquired on an axial plane before contrast-agent injection, and DWI with *b* values of 50, 500, and 800 s/mm${}^{2}$ was acquired on the axial plane with their corresponding ADC maps. DCE-MRI studies were obtained with intravenous administration of paramagnetic contrast agent (Prohance, Bracco Imaging, Italy) 0.3 mmol/kg, a flow rate of 3.5 mL/s, injected after six pre-contrast transaxial T1 Vibe with flip angles of 2${}^{\circ }$, 5${}^{\circ }$, 8${}^{\circ }$, 12${}^{\circ }$,15${}^{\circ }$, and 20°, followed by a T1 Vibe axial dynamic (TR/TE = 5.47/1.75) with 60 measurements over a 10 min period and a temporal resolution of 9.6 s. Subtracted DCE images (SUB) were obtained automatically by subtracting pre-contrast images from the post-contrast (PC) images. Finally, an axial high-resolution T1 Vibe with fat suppression (HR Vibe T1-w fat sat) was acquired. Technical details of MRI sequences are shown in Table 1.

### 2.4. Image Processing and 3D ROI Segmentation

**Figure 2:**
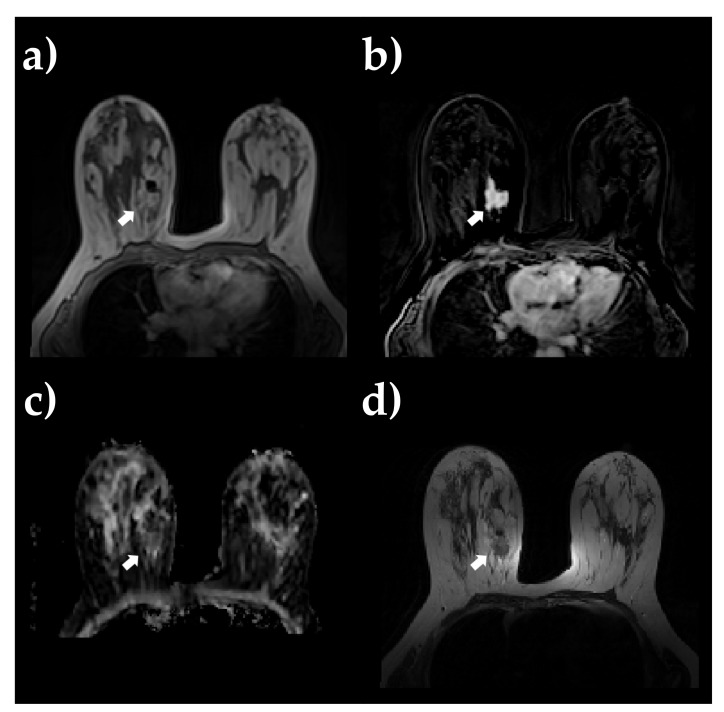
Example of primary BC lesion shown on a pretreatment breast MRI: (**a**) post-contrast T1 images, (**b**) DCE-MRI subtraction images with the highest mean signal intensity within the ROI, (**c**) ADC map, and (**d**) T2 images.

ADC images were non-rigidly coregistered on SUB PC DCE-MRI images using Elastix software (v4.9.0) to correct for typical spatial distortion arising from DWI acquisition. T2 images were all resliced on DCE-MRI images. Lesion segmentation was performed on SUB DCE images by an experienced radiologist using an in-house developed software for region labeling. During the segmentation procedure, the radiologist was blinded to both the histological results and all clinical information relative to the retrospective breast mpMRI images. The delineated ROIs were then copied and pasted into the PC DCE-MRI, registered ADC, and resliced T2 images (refer to Figure 2 for an example of primary BC lesion). Before radiomic feature extraction, normalization was applied on T2 and PC image intensities. Specifically, intensities were normalized by centering them at their respective mean value with a standard deviation of all grey values in the original image [25].

### 2.5. Feature Extraction

The extraction of radiomic features from 3D Regions of Interest (ROIs) on DCE-MRI subtraction series with the highest mean signal intensity within the ROI [26,27,28], PC DCE-MRI, registered ADC, and resliced T2 images was performed using the open source PyRadiomics package [29]. The obtained features can be classified into five classes: (i) shape features (n = 14); (ii) first-order features (n = 18); (iii) 73 s-order textural statistics including grey-level co-occurrence matrix (GLCM) (n = 24), grey-level run length matrix (GLRLM) (n = 16), grey-level size zone matrix (GLSZM) (n = 16), neighboring grey tone difference matrix (NGTDM) (n = 5), and grey-level dependence matrix (GLDM) (n = 14); 1092 transformed first-order and textural features including (iv) 728 wavelet features in frequency channels LHL, LLH, HHH, HLH, HLL, HHL, LHH, and LLL, where L and H are low- and high-pass filters, respectively; and (v) 364 Laplacian of Gaussian filtered features with sigma ranging from 2.0 to 5.0, with a step size = 1.

### 2.6. Two-Step Feature Selection and Learning

Since the number of extracted radiomics features was very high, using all of them for the classification step was generally ineffective because these features are redundant and highly correlated. Moreover, when the number of features is much higher than the number of samples, the classification process might yield low-quality results due to the so-called curse of dimensionality. Thus, a feature selection process has been applied to remove redundancies while preserving features that might give greater contributions in terms of classification [30].

Seven feature selection methods, described in Table 2, were used. These techniques were chosen mainly because of their popularity in literature, simplicity, and computational efficiency [31]. Table 2 classifies the methods based on their type, i.e., ranker or subset, relation with the subsequent classification approach, and returned results [32].

Before the classification step, the Synthetic Minority Oversampling Technique (SMOTE) algorithm [33] was applied to overcome the over-fitting problem that might arise when an unbalanced set of data is used. New samples in feature space were produced through data interpolation among the instances that lie together, obtaining a more balanced set. Finally, to perform classification, six well-known ML algorithms have been exploited [34]: K-Nearest Neighbors (KNN) [35], Naive Bayes (NB) [36], Support Vector Machine (SVM) [37], Decision Tree (DT) [38], Multi-Layer Perceptron (MLP) [39], and Random Forest (RF) [40].

There is not a universally recognized ideal approach that could be considered as a standard choice for feature selection: indeed, different methods, combined with various classification algorithms, might give very different results on the same dataset, as well as in terms of the generalization ability of the extracted feature subset on new data. Moreover, the datasets examined in this work were characterized by a small number of patients with respect to the number of available features. To better exploit the available data, a cross-fold validation approach was chosen rather than dividing the dataset into the training, validation, and test set. This choice, in turn, raised the problem of merging the features selected over the different folds.

**Figure 3:**
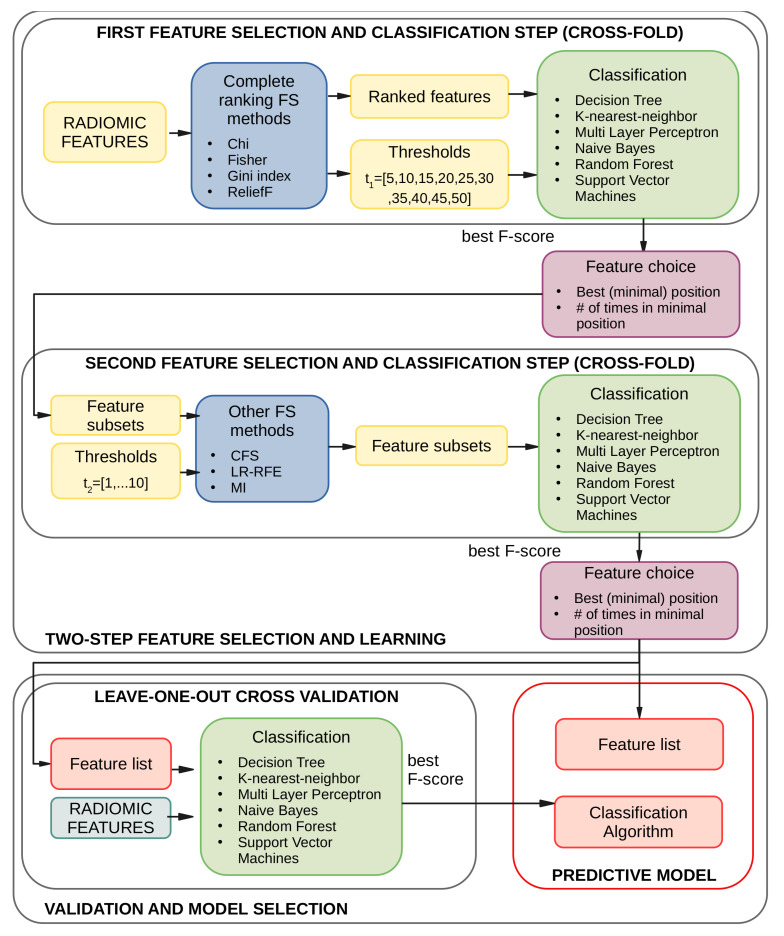
A schematic view of the ensemble feature selection and classification steps underlying the building process of each predictive model. The first two steps involve 10-fold cross-validation on the training dataset. The last step is based on a leave-one-out cross-validation (LOOCV) approach to validate the combination of selected features and classification models. The best F-score values drive the choice of the classification algorithm at each step.

To address the problem, a two-step approach was adopted: in the first step, complete filter methods were exploited to greatly reduce the amount of features used for classification, whereas, in the second step, more complex algorithms were employed to fine-tune the selection of the most representative features. This process was inspired by similar methodologies, such as those proposed by Ge et al. and Yang et al. [41,42], designed to address the same issue of having a higher number of features than the input samples, a very typical condition when dealing with biomedical data. In the first step of the pipeline, the complete ranker filter methods (Chi Squared, Fisher Score, Gini Index, and ReliefF) were used to delete the most redundant features. In the second step, three different approaches were considered to boost diversity: i) the Least Absolute Shrinkage and Selection Operator (LASSO) Regression with Recursive Feature Elimination (LR-RFE), the Mutual Information (MI) method, and Correlation-based Feature Selection (CFS). The proposed two-step feature selection pipeline is fully described in Figure 3. In the first learning step, the complete ranking methods were applied on all the extracted folds and using a predefined range of feature numbers set by the threshold $t_{1}$. The highest classification performance, expressed in terms of F1-score, was evaluated among all the feature selection and classification method combinations and for the different thresholds $t_{1}$. Then, the associated feature subset must be combined over the different folds used for the classification process. Similar to homogeneous ensemble learning, in which solutions belonging to different data splits are combined, the feature selected over the folds must be aggregated to produce a final reference set. Following Bolon et al. [43], an aggregation strategy based on tracking the minimal rank of each feature ($min_{pos}$) and the number of times it has been chosen in that position ($num_{pos}$) over the different folds was used. The features were ordered by $min_{pos}$ first and $num_{pos}$ after, thus obtaining a list of ordered features over all the folds. The features reaching the position specified by the threshold $t_{1}$ at least once were selected and became the new feature set on which the second step of the pipeline was then performed. The strategy was very similar to the previous step, with the slight difference that the number of the extracted features had to be directly specified to the selection algorithms (except for CFS, in which the number was automatically determined). Once again, the features were ordered as described above, but they were filtered using a lower threshold $t_{2}$. It is worth noting that the defined thresholds were applied on the minimum position reached: the final amount of features selected might be greater than the threshold itself, owing to the different features extracted across the folds in the validation steps.

### 2.7. Model Selection and Validation

At the end of the two-step learning phase, the list of the most relevant features was obtained. Since the dataset size did not allow for a separate test set, and an external dataset with the same characteristics (e.g., same image type and modalities, same annotation markers) was missing, an LOOCV approach was applied to the original dataset to build the predictors. Hence, to further validate the results and simultaneously determine the best classification algorithm, a final step was performed using all the classification algorithms. The final feature list and the chosen classification algorithm constituted the final predictor. The bottom section of Figure 3 depicts this last step.

To assess the generalization abilities of the models and, more generally, the validity of the proposed two-step approach, the predictors were tested on the DS${}_{T}$.Unfortunately, only the predictors for the ER and PR markers can be tested since the available information regarding HER2 was partial or totally missing as in the case of Ki67. In addition, only the T1 and T2 images were present, whereas the ADC and SUB were unavailable. We used the same approach described above to assign samples to the classes whenever the needed information was available and the positive/negative official label otherwise. We ended up with 91 patients for both the ER and PC markers, with the classes distributed as follows: ER−/ER+ = (15.4%/84.6%) and PR−/PR+ = (20.9%/79.1%).

Therefore, only the ER and PR detection tests have been performed using the feature subset selected in the last step of the pipeline.

## 3. Results

### 3.1. Experiments and Settings

The proposed pipeline has been applied separately to the four molecular markers. Data from the DS${}_{M}$ comply with the common practice in markers annotation, with ER, Ki67, and PR expressed in percentages (of involved cells), whereas the HER2 had discrete values followed, in this case, by one or more plus signs. To train the classification models, the markers’ expression values were binarized according to the following criteria: the ER and PR markers were considered negative if their value was lower than 10% and positive otherwise; the Ki67 marker was considered negative if it had a value lower than 14% and positive otherwise; and HER2 was considered negative if its value was 0 or 1 and positive otherwise [44]. The distribution of data classes obtained following those criteria is reported in Table 3. Except for the PR marker, the class distribution is strongly unbalanced. In the two-step feature classification process, the threshold $t_{1}$, which should provide a coarse-grained feature subset, was set to $t_{1}=\left[5,10,15,...,50\right]$, whereas the $t_{2}$ threshold was used to extract a fine-grained feature subset and was set to $t_{2}=\left[1,...,10\right]$. These values were selected for: (i) having a comparable number of input samples and features in the classification steps and (ii) trying to extract a smaller, meaningful representative subset able to generalize across datasets. Due to the reduced number of input samples, a 10-fold cross-validation approach was used in the first two steps of the feature selection pipeline. Generally, when working on classification models in which the dataset is unbalanced, the F1-score, which combines precision and recall into a single metric, is a suitable measure. Thus, the F1-score was used to select the best training models. In the next subsections, the results of the first and second steps of the feature selection are reported separately. Moreover, different model settings were taken into account:

- Radiomics from a single MRI sequence;
- Radiomics from all MRI sequences;
- Radiomics from a ingle MRI sequence + clinical information (i.e., patient’s age);
- Radiomics from all MRI sequences + clinical information (i.e., patient’s age).

Regarding implementation details, both classifiers and feature selection algorithms have been implemented in Python 3.7, using the Scikit-learn framework [45] and scikit-feature package [46], respectively. As for the over-sampling algorithm SMOTE, we adopted the implementation provided by the Imbalanced-learn library [47].

### 3.2. Results of the First Feature Selection Step

All the experiments performed for the first step of the feature selection are available in the Supplementary Materials (Tables S1–S4). However, to give the reader an idea of the available information, some results are reported in Table 4. For each of the different input combinations, the best F1-score is shown. Features from different image modalities were taken all together or used separately. Considering the ER marker, the T2 modality emerged as the one performing better when using only radiomics features and also with a combination of radiomics and clinical data. When all the image modalities were used together, the performances decreased. Considering the HER2 marker, ADC was the modality giving the best results, whereas for Ki67, the best performances were obtained using all the image modalities and only the radiomics features. Finally, for the PR marker, the PC modality gave the best results both with clinical data and without them.

Referring to the Supplementary Tables S1–S4, the classification results obtained with the complete set of features were generally much worse than the ones obtained after the feature selection. This result was expected since the number of features was much higher than the number of input data. Hence, feature selection was confirmed to be a mandatory step when performing classification in these conditions.

The Supplementary Materials show the results obtained for all the possible combinations of features, namely those using all the radiomics modalities (thus totaling more than four thousand features for each patient), using both the radiomics and the clinical information, and using single radiomics modalities (about one thousand features for each patient). The two best results were considered for each feature combination, and the feature threshold and selection algorithm used for each of them was reported. On the feature subsets obtained from the two best results, the second pipeline step was then applied. In all the cases, the only clinical feature available (that is, the patient’s age) did not emerge as an important one. Hence, only the radiomics features were used. There is no preferred feature selection algorithm over the four biomarkers and the different feature combinations. The Fisher Score method was one of the most used, thus suggesting that it could be a good starting choice if one needs to perform a single feature selection step. When the imaging modalities were used together, the ReliefF method generally gave the best results due to its higher robustness to noise and redundancy. Nonetheless, both for the HER2 and Ki67 markers, on which ReliefF and Fisher Score gave the best results, the subsequent feature selection step performs better when using the second best (i.e., Gini Index for HER2 and Chi Squared method for Ki67, as reported in the following subsection). This might be due to the classification algorithm overfitting the selected features owing to the small dataset size. Regarding the radiomics modalities, the SUB one always gave poor results, whereas the T2 and PC were often preferred. The ADC alone never emerged, but it proved useful in combination with other modalities. In addition, for the $t_{1}$ threshold, there is no preferred value. The $t_{1}$ value giving the best results changes widely along the different modalities, features, and classification algorithms.

### 3.3. Results of the Second Feature Selection Step

The final classification results for all the markers obtained after the second feature selection step are shown in terms of F1-score in Table 5 and in terms of accuracy, precision, and recall in Table 6. In addition, in the second pipeline step, there was no clearly winning feature selection algorithm. LR-RFE and CFS gave the best results, with the MI approach performing generally worse. MLP is the classification algorithm more frequently used, although high results were also obtained with the DT and the KNN models (for the HER2 and PR markers, respectively). In the final step, the best-performing classification algorithm was chosen to obtain the final predictor. There was no preferred one, with MLP usually performing as well as the SVM, with the second being more prone to overfitting or learning a single class in the most unbalanced cases (as in Ki67).

**Figure 4:**
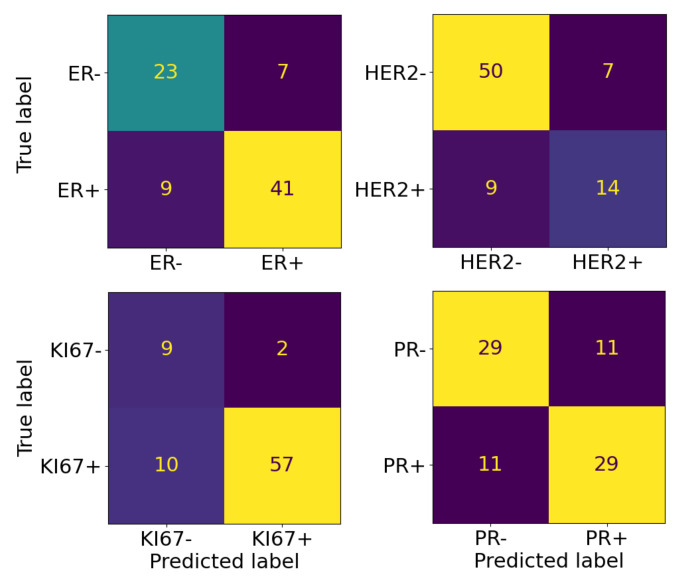
Confusion matrix for the four markers at the end of the two-step pipeline.

The F1-scores for the LOOCV step were calculated considering the two labels in turn as the positive class. Indeed, for these markers, both the negative and positive states are important in defining the cancer’s molecular subtype. Especially in the case of the HER2 marker, whose class distribution was highly unbalanced, with 71% of samples belonging to the negative class, it was very important to understand how the predictor behaved. Being trained on a majority of negative samples, it performed better in detecting negative samples. However, it had good abilities also with a positive sample. When the classes were balanced, as in the PR case, there was no difference between the two values. Confusion matrices associated with performances of the predictors at the end of the two-step pipeline were reported in Figure 4.

The final feature number was obtained in the LOOCV step, and the list of the extracted features list is available in Table 7. For HER2, the best results were obtained with a combination of features coming from two different image modalities (i.e., ADC, T2). For the Ki67 and PR markers, the features all belong to the PC modality, whereas for the ER marker, they belong to the T2 one.

**Figure 5:**
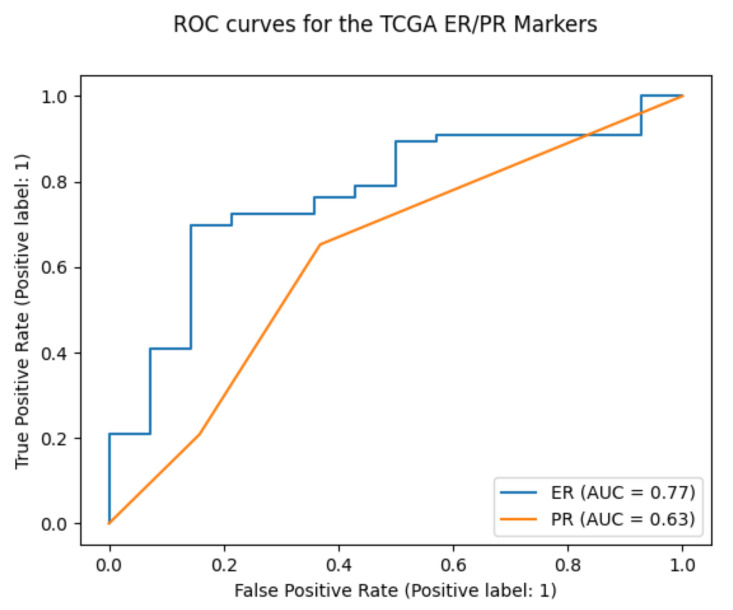
ROC curves and Area Under the Curve (AUC) for the ER and PR markers on the DS${}_{T}$ validation dataset.

Concerning the validation performed on the DS${}_{T}$, a noteworthy F1-score of 0.88 was obtained for the ER marker. Since other works report their performances based on the Area Under the ROC curve (AUC), this measure was also computed for the ER marker, obtaining an AUC = 0.77. For the PR marker, results were less encouraging. The F1-score was 0.88, but only a single class (the positive one) was predicted on those data, thus resulting in an AUC = 0.63, as expected for this condition. Figure 5 shows the curves obtained for the two markers.

## 4. Discussion

This study aimed at comparing the performance of different radiomic models in the prediction of the four most widely used molecular markers (ER, HER2, Ki67, PR) in BC management, using a two-step feature selection radiomic approach to extract meaningful mpMRI feature subsets. Predictions were performed under different settings, i.e., with or without clinical features with radiomics and, for the latter, in all the mono- and multi-modality combinations of MRI sequences.

The results obtained in this study demonstrated that the proposed approach was an accurate method to pre-operatively predict the most relevant molecular markers, with the best resulting models composed of only radiomic features and reaching F1-scores up to 0.9. In particular, for HER2, the best results were obtained with an SVM model built with three T2 texture features and the minimum value of ADC from the LLH wavelet transformed ADC map. Other studies found that MRI-based features were associated with the HER2 status of patients with BC [48]. For the Ki67 and PR markers, the best results were obtained with an MLP model built with features all belonging to the PC modality, which currently represents the clinical standard for characterizing BC lesions [49]. These results are partially in accordance with some previous studies investigating the power of radiomics for Ki67 and PR status prediction [50,51,52], although they also found promising results arising from different sequences. Of note, shape flatness was the only shape feature that contributed both in the PR prediction model and in the best-performing model for the ER marker (RF), which was surprisingly built entirely on T2 features. Shape flatness characterizes the shape of the tumor, and in particular, a small flatness value indicates an irregular tumor shape. This feature has been shown to have power in the prognostic prediction of BC patients [53,54]. It is worth noting that, except for the prediction of HER2+ status, radiomics features are critical for model construction derived from a single MRI sequence.

From the methodological point of view, our two-step pipeline is novel, although it shares some similarities with approaches used in other works. In particular, in Xie et al. [55], a two-step pipeline was proposed to filter features, distinguishing between coarse-grained and fine-grained feature subsets with the classification target of simultaneously classifying the four immunohistochemically derived cancer subtypes. They reported a mean accuracy of 0.72 on a private dataset. Apart from being different in the imaging modalities and the learning targets used, they relied on a single statistical method for the first feature selection step, whereas we exploited several different algorithms to choose the most suitable one for the problem at hand. We suggested the importance of exploiting features coming from different imaging modalities, as also reported in Liu et al. [56], where they use conventional T2, DWI, and T1w DCE imaging to predict cancer subgroups and in particular to distinguish between HER2-positive/negative receptor status. They evaluated the performances on a private dataset and reported good results in the training phase (AUC = 0.78) and lower in the testing phase (AUC = 0.62), with better performances for the models exploiting multimodal features than monomodal ones. Notably, we found that for HER2, a multimodal feature set is needed to classify patients properly. This intuition was confirmed by the drop in F1-score to 0.57 when the ADC feature is removed. This result also underlines the critical importance of DWI for BC characterization, as also reported in previous studies [57].

Unfortunately, it was not possible to directly compare the results obtained with the proposed methodology with similar strategies on the same learning target, owing to the different, not publicly available dataset used. However some information could be extracted by looking at large-scale studies such as [58]: the authors exploited feature selection and machine learning (ML) approaches on a large private DCE-MRI dataset, obtaining an AUC = 0.65 for the ER status using training and test sets extracted from the same dataset. The result obtained in this study was higher, suggesting the good generalization abilities of the proposed predictor. In Li et al. [19], about forty radiomics features were extracted from the same TCGA-BRCA dataset we used, and the prediction ability of the features was assessed on the four clinical biomarkers through statistical analyses. Considering the ER biomarker prediction, they reached an AUC = 0.89. In Guo et al. [14] the authors used logistic regression to predict different outcomes, including the ER marker on which they obtained an AUC = 0.79. Again, a direct comparison was not possible since the predictors were built directly on the TCGA-BRCA data, while in this work, this dataset was only used as a test set (DS${}_{T}$). However, it is essential to emphasize that the relevance of the obtained result lies in having the models trained on a dataset completely different from the one used to test them. Concerning the PR marker, the results from the already cited works reported AUCs of 0.62, 0.69, and 0.69, respectively. In this study, the predictor was not able to distinguish among the PR− /PR+ classes and assigned all the patients to the PR+ class. This was somehow expected given the differences between the used datasets and the small size of the training data and deserves to be further explored with the usage of additional data for the training phase. Referring to the Supplementary Materials, Table S5 reports a comparison of the experimental design adopted by the aforementioned works.

Despite the interesting results obtained, this study suffers from some limitations. First, the sample size for the analysis was small and, except for the PR+/PR− classification task, unbalanced. A larger and more balanced study group is needed to perform a better radiomic analysis and build more robust prediction models. Although the model’s performance was corrected by using 10-fold CV in the main classification step and LOOCV for the building of prediction models, such imbalance might have influenced the development of the ML model and the results [59]. Second, the study was retrospective and needed to be validated with other comprehensive external cohorts to determine the value of the developed model in clinical practice and improve the confidence of performance. Furthermore, prospective and multicentric studies need to be performed to define a potential standardization of the proposed approach. Moreover, the lack of standardization in radiomic investigations, in terms of image acquisition, processes, segmentation methods, and radiomics analysis tools, could lead to discrepancies in radiomic feature measurements that are not due to underlying biological variations.

Reproducibility of radiomic features is of crucial importance to clinical applications in the field of BC [60]. Of note, to extract radiomic features, we used the PyRadiomics software [61] which: (i) is compliant with the Image Biomarker Standardization Initiative (IBSI) guidelines that promoted the standardization of radiomic analysis [62], (ii) allows for a reproducible extraction of radiomic features due to the parameter files that could be shared and re-used, and (iii) can also be used starting from DICOM input images with the file name pointing to a DICOM Segmentation Image object, thus automatically obtaining radiomic features without any intermediate steps. This choice allows for a reproducible feature extraction under real clinical conditions that usually involve DICOM objects [27]. In addition, according to Lambin et al. [63], a detailed report of all the steps of the radiomic workflow performed in the study was carried out to improve both clinical translation in this emerging field and the reproducibility of study outcomes. Another limitation affecting this study concerned the use of manual segmentation for the VOIs’ delineation, which is time- and labor-consuming and prone to user variability. More accurate and automatic tumor segmentation tools are needed to improve the quality of the radiomic analysis in future works. On a positive note, in this study, 3D ROIs were used for lesion segmentation. The aim was to decrease inter-reader variability by eliminating the requirement to choose a single-slice corresponding to a portion of a lesion. Hence, a more thorough description of the lesion is obtained by an increase in the number of points considered for feature computation, which improved the accuracy of characterization of heterogeneous lesions and lowered the sampling errors [64].

## 5. Conclusions

The MRI-based radiomic approach developed in this work, built on a comprehensive BC dataset including MRI sequences and molecular outcomes, can efficiently characterize BC heterogeneity according to the most examined biomarkers (ER, PR, HER2, and Ki67). This methodology might be of great support for BC management for the following reasons: (i) it has the advantage of being developed on an appropriate two-step feature selection and classification technique; (ii) it implements an effective comparison of different models and feature selection approaches); (iii) it is externally validated whenever possible; and (iv) it addresses the well-known issues arising from the lack of available BC datasets by exploiting a comprehensive dataset of molecular markers and MRI modalities. Moreover, the developed two-step pipeline is general enough to be used on similar classification problems on different cancer types. Our results also highlighted the potential and strength of using only mpMRI data for high-quality BC radiomics analysis. Further prospective and multicentric studies need to be performed to define a potential standardization of our approach. In the future, larger BC cohorts will be investigated to validate our results more extensively.

## Figures and Tables

**Table 1:** Technical details of acquired MRI sequences. TR = Repetition Time; TE = Time to Echo; FA = Flip Angle; ST = Slice Thickness; FOV = Field Of View; Aver. = average; Meas. = measurements.

Sequence	TR (ms)	TE (ms)	FA (${}^{\circ }$)	Slices	ST (mm)	Voxel Size	Matrix	FOV (mm)	Aver.	Meas.	Time (min)	b-Value (s/mm${}^{2}$)
TSE T2	5440	81	80	40	4.0	0.8 × 0.8	448	340	2	-	03:34	-
DWI ax	9600	74	90	25	4.0	1.8 × 1.8	192	340	3	-	04:48	50/500/800
DCE	5.47	1.75	20	36 (slab1)	3.6	1.7 × 1.7	192	320	1	60	09:39	-
HR Vibe T1-w fat sat	8.69	4.33	15	176 (slab1)	0.9	0.8 × 0.8	448	340	1	-	03:21	-

**Table 2:** List of the feature selection methods used, with a brief classification of the type, approach, and returned results. LR-RFE: Lasso Regression with Recursive Feature Elimination; CFS: Correlation-based Feature Selection.

Algorithm	Type (Ranker/Subset)	Approach (Filter/Wrapper)	Result (Complete/Partial)
Chi Squared	Ranker	Filter	Complete
Fisher Score	Ranker	Filter	Complete
Gini Index	Ranker	Filter	Complete
Mutual Information	Ranker	Filter	Partial
ReliefF	Ranker	Filter	Complete
LR-RFE	Ranker	Wrapper	Partial
CFS	Subset	Filter	Partial

**Table 3:** Thresholds used to define the DS${}_{M}$ positive/negative classes, and the derived distribution of the samples among them.

Marker Name	Total Samples	Positive Threshold	Sample Class # (%)	
			Negative	Positive
ER	80	≥10%	30(37.5%)	50(62.5%)
HER2	80	≥2	57(71%)	23(29%)
Ki67	78	≥14%	11(14%)	67(86%)
PR	80	≥10%	40(50%)	40(50%)

**Table 4:** Best results of the first feature selection step for all markers. For each combination of features (i.e., single radiomics, single radiomics with clinical information) and for each combination of modalities (i.e., all together (ALL) and single), the best mean F1-score obtained over the folds is shown. Bold font indicates the best results for each marker.

Marker	Feature Type	Image Modality	Feature Selection Algorithm	Feature Threshold t1	F1-Score ± Variance
ER	**radiomics from** **single modalities**	**T2**	**fisher**	**45**	**0.69 ± 0.02**
	radiomics from single modalities/clinical	T2	fisher	25	0.68±0.01
	radiomics/clinical	ALL	chi	50	0.65±0.04
	radiomics	ALL	chi	25	0.59±0.04
HER2	**radiomics from** **single modalities**	**ADC**	**reliefF**	**30**	**0.7 ± 0.03**
	radiomics from single modalities/clinical	ADC	reliefF	10	0.69±0.03
	radiomics/clinical	ALL	gini index	10	0.68±0.02
	radiomics	ALL	reliefF	30	0.62±0.02
Ki67	radiomics from single modalities	PC	chi	10	0.77±0.06
	radiomics from single modalities/clinical	PC	gini index	5	0.75±0.05
	radiomics/clinical	ALL	chi	50	0.72±0.03
	**radiomics**	**ALL**	**fisher**	**20**	**0.79 ± 0.05**
PR	**radiomics from** **single modalities**	**PC**	**fisher**	**5**	**0.73 ± 0.03**
	**radiomics from** **single modalities/clinical**	**PC**	**fisher**	**5**	**0.73 ± 0.03**
	radiomics/clinical	ALL	reliefF	15	0.67±0.07
	radiomics	ALL	reliefF	15	0.67±0.07

**Table 5:** Results for the four markers after the proposed two-step pipeline and the model validation and selection. The features and the learning algorithm obtained at the final LOOCV step are used to build the model predictors. Only the best result (in terms of F1-score) is shown here for each marker. Since the classes were unbalanced (except for the PR marker), the F1-score obtained considering both the label values as the positive class is reported. FSA: Feature Selection Algorithm; LA: Learning Algorithm.

Best Training Results										
Marker Name	1st Step Results				2nd Step Results			LOO Results		
	Feat. Type	FSA	$t_{1}$	**F1-Score** ±**var**	FSA	$t_{2}$	F1-Score ±var	LA	Feat. #	F1-Score pos/neg
ER	T2	fisher	45	0.69±0.02	lr rfe	10	0.72±0.01	svm	11	0.85/0.81
HER2	ALL	gini	10	0.68±0.02	cfs	5	0.75±0.04	rf	5	0.64/0.86
Ki67	PC	chi	10	0.77±0.06	cfs	1	0.79±0.05	mlp	2	0.9/0.84
PR	PC	fisher	5	0.73±0.03	lr rfe	3	0.74±0.03	mlp	3	0.73/0.73

**Table 6:** Results for the four markers in terms of other metrics: acc = accuracy; prec = precision; and rec = recall.

Best Training Results (Other Metrics)									
Marker Name	1st Step Result			2nd Step Result			LOO Result		
	acc ± var	prec ± var	rec ± var	acc ± var	prec ± var	rec ± var	acc	prec	rec
ER	0.73 ± 0.02	0.72 ± 0.02	0.7 ± 0.02	0.74 ± 0.01	0.74 ± 0.02	0.72 ± 0.01	0.81	0.87	0.82
HER2	0.73 ± 0.02	0.71 ± 0.02	0.71 ± 0.02	0.8 ± 0.02	0.75 ± 0.04	0.77 ± 0.04	0.8	0.67	0.61
KI67	0.89 ± 0.01	0.76 ± 0.06	0.8 ± 0.06	0.87 ± 0.02	0.78 ± 0.05	0.84 ± 0.05	0.85	0.97	0.85
PR	0.74 ± 0.03	0.77 ± 0.03	0.74 ± 0.03	0.75 ± 0.02	0.79 ± 0.02	0.75 ± 0.02	0.73	0.73	0.73

**Table 7:** Selected features at the end of the two-step pipeline for the four biomarkers.

ER
original_shape_Flatness
T2_original_glcm_Idmn
T2_wavelet-LHH_glszm_ZoneEntropy
T2_wavelet-LHH_gldm_LargeDependenceLowGrayLevelEmphasis
T2_wavelet-LLH_glszm_SmallAreaLowGrayLevelEmphasis
T2_wavelet-LLH_gldm_SmallDependenceLowGrayLevelEmphasis
T2_wavelet-LLH_firstorder_Skewness
T2_log-sigma-4-0-mm-3D_glcm_Imc2
T2_log-sigma-4-0-mm-3D_firstorder_Skewness
T2_log-sigma-5-0-mm-3D_glszm_SmallAreaEmphasis
T2_log-sigma-5-0-mm-3D_firstorder_Skewness
**HER2**
T2_original_glrlm_RunVariance
T2_original_glrlm_LongRunEmphasis
T2_original_glszm_ZonePercentage
T2_original_gldm_DependenceNonUniformityNormalized
ADC_wavelet-LLH_firstorder_Minimum
**Ki67**
PC_wavelet-LHH_gldm_DependenceEntropy
PC_wavelet-HHL_gldm_SmallDependenceLowGrayLevelEmphasis
**PR**
original_shape_Flatness
PC_wavelet-LLH_glcm_Correlation
PC_wavelet-HHH_ngtdm_Busyness

## Data Availability

The DS${}_{M}$ used in this paper come from the MOBL−OB collection of BCU Imaging Biobank, available under request.

## Supplementary Materials

The following are available online at https://www.mdpi.com/article/10.3390/s23031552/s1, Tables S1–S4: Results of the first and second feature selection steps and of the final classification for the tasks ER+/ER−, HER2+/HER2−,KI67+/KI67−, PR+/PR2−; Table S5: Retrospective studies on predicting BC molecular subtypes.

## Abbreviations

The following abbreviations are used in this manuscript:

ADC	Apparent Diffusion Coefficient
AUC	Area Under Receiver Operator Characteristic (ROC) Curve
BC	Breast Cancer
CFS	Correlation-based Feature Selection
CNN	Convolutional Neural Network
DCE	Dynamic Contrast-Enhanced
DICOM	Digital Imaging and COmmunications in Medicine
DWI	Diffusion Weighted Imaging
DT	Decision Tree
ER	Estrogen Receptor
FSA	Feature Selection Algorithm
GLCM	Gray-Level Co-occurrence Matrix
GLDM	Gray-Level Dependence Matrix
GLRLM	Gray-Level Run-Length Matrix
GLSZM	Gray-Level Size Zone Matrix
HER2	Human Epidermal growth factor Receptor
IBSI	Image Biomarker Standardization Initiative
KNN	K-Nearest Neighbors
LA	Learning Algorithm
LASSO	Least Absolute Shrinkage and Selection Operator
LOOCV	Leave-One-Out Cross-Validation
LR	LASSO Regression
MI	Mutual Information
ML	Machine Learning
MLP	Multi Layer Perceptron
mpMRI	multiparametric MRI
MRI	Magnetic Resonance Imaging
NB	Naive Bayes
NIfTI	Neuroimaging Informatics Technology Initiative
NGTDM	Neighbouring Gray-Tone Difference Matrix
PC	Post Contrast
PR	Progesterone Receptor
RF	Random Forest
RFE	Recursive Features Elimination
ROC	Receiver Operator Characteristic
ROI	Region Of Interest
SUB	Subtracted
SVM	Support Vector Machine
SMOTE	Synthetic Minority Oversampling TEchnique
T2	T2-weighted
TCIA	The Cancer Imaging Archive
TCGA-BRCA	The Cancer Genome Atlas BReast Invasive CArcinoma
