# Supplementary material for: A Two-Step Feature Selection Radiomic Approach to Predict Molecular Outcomes in Breast Cancer
Source: Sensors (Basel). 2023 Jan 31;23(3):1552. doi: 10.3390/s23031552 (PMC9921618; doi:10.3390/s23031552)
Supplement: Supplementary file 1 [file sensors-23-01552-s001.zip › sensors-2029658-supplementary.pdf]

Table S1.

Results of the first and second feature selection steps (first two sub-tables), and of the final classification (last sub-table) for the ER+/ER- classification task.

Positive/Negative ER marker. Number of patients=80 (P=62.5%,N=37.5%). First feature selection step						
**Feature type**	**Image modality**	**Algorithm**	**Feature selection algorithm**	**Feature threshold t1**	**accuracy ± variance**	**f1score ± variance**
**radiomics from single modalities**	**ADC**	svm	no_alg	all	0.56 ± 0.02	0.53 ± 0.03
		mlp	fisher	45	0.61 ± 0.03	0.58 ± 0.02
		rf	fisher	45	0.6 ± 0.03	0.56 ± 0.03
	**PC**	dt	no_alg	all	0.64 ± 0.01	0.6 ± 0.01
		dt	reliefF	20	0.65 ± 0.03	0.62 ± 0.04
		svm	reliefF	5	0.61 ± 0.02	0.6 ± 0.02
	**SUB**	dt	no_alg	all	0.53 ± 0.03	0.5 ± 0.03
		mlp	reliefF	20	0.63 ± 0.05	0.59 ± 0.06
		knn	gini_index	5	0.6 ± 0.02	0.59 ± 0.02
	**T2**	mlp	no_alg	all	0.7 ± 0.04	0.66 ± 0.05
		**mlp**	**fisher**	**45**	**0.73 ± 0.02**	**0.69 ± 0.02**
		mlp	fisher	35	0.7 ± 0.04	0.67 ± 0.04
**radiomics from single modalities/clinical**	**ADC**	rf	no_alg	all	0.59 ± 0.03	0.54 ± 0.03
		dt	gini_index	30	0.6 ± 0.03	0.58 ± 0.04
		mlp	chi	50	0.61 ± 0.03	0.57 ± 0.03
	**PC**	dt	no_alg	all	0.58 ± 0.02	0.5 ± 0.03
		dt	chi	50	0.65 ± 0.03	0.62 ± 0.04
		dt	reliefF	15	0.66 ± 0.02	0.62 ± 0.02
	**SUB**	rf	no_alg	all	0.63 ± 0.01	0.59 ± 0.01
		dt	reliefF	5	0.66 ± 0.07	0.65 ± 0.07
		mlp	chi	25	0.63 ± 0.05	0.6 ± 0.05
	**T2**	mlp	no_alg	all	0.69 ± 0.02	0.65 ± 0.03
		**mlp**	**fisher**	**25**	**0.7 ± 0.01**	**0.68 ± 0.01**
		mlp	chi	35	0.7 ± 0.03	0.66 ± 0.04
**radiomics/clinical**	**ALL**	mlp	no_alg	all	0.56 ± 0.04	0.51 ± 0.05
		**mlp**	**chi**	**50**	**0.68 ± 0.04**	**0.65 ± 0.04**
		svm	chi	50	0.61 ± 0.06	0.61 ± 0.06
**radiomics**	**ALL**	mlp	no_alg	all	0.58 ± 0.05	0.53 ± 0.06
		**mlp**	**chi**	**25**	**0.63 ± 0.03**	**0.59 ± 0.04**
		mlp	reliefF	15	0.63 ± 0.02	0.59 ± 0.02

Second feature selection step						
**Feature type**	**Image modality**	**Algorithm**	**Feature selection algorithm**	**Feature threshold t2**	**accuracy ± variance**	**f1score ± variance**
**radiomics from single modalities**	**T2**	**mlp**	**lr**	**11**	**0.74 ± 0.01**	**0.72 ± 0.01**
		mlp	lr	43	0.74 ± 0.03	0.71 ± 0.04
**radiomics from single modalities/clinical ***	**T2**	mlp	lr	9	0.73 ± 0.02	0.68 ± 0.03

*only radiomics were selected

Predictor building with the Leave-One-Out approach						
**Feature type**	**Image modality**	**Algorithm**	**Feature selection algorithm**	**Feature number**	**accuracy**	**f1score**
**radiomics from single modalities**	**T2**	**svm**	no_alg	**11**	**0,81**	**0,85**
		mlp	no_alg	11	0,8	0,84

in **bold** = best result (F1score) for each “Feature type” section
in **red** = best result (F1score) among ALL the “Feature type” sections

Table S2.

Results of the first and second feature selection steps (first two sub-tables), and of the final classification (last sub-table) for the HER2+/HER2- classification task.

Positive/Negative HER2 marker. Number of patients=80 (P=29%,N=71%) First feature selection step						
**Feature type**	**Image modality**	**Algorithm**	**Feature selection algorithm**	**Feature threshold t1**	**accuracy ± variance**	**f1score ± variance**
**radiomics from single modalities**	**ADC**	dt	no_alg	all	0.64 ± 0.02	0.56 ± 0.02
		**rf**	**reliefF**	**30**	**0.76 ± 0.01**	**0.7 ± 0.03**
		mlp	gini_index	5	0.71 ± 0.03	0.65 ± 0.05
	**PC**	nb	no_alg	all	0.66 ± 0.02	0.52 ± 0.03
		svm	reliefF	5	0.66 ± 0.03	0.62 ± 0.04
		mlp	fisher	15	0.64 ± 0.04	0.58 ± 0.06
	**SUB**	nb	no_alg	all	0.71 ± 0.03	0.58 ± 0.07
		knn	fisher	5	0.7 ± 0.04	0.67 ± 0.04
		dt	fisher	15	0.69 ± 0.02	0.62 ± 0.03
	**T2**	mlp	no_alg	all	0.68 ± 0.03	0.59 ± 0.03
		dt	gini_index	40	0.7 ± 0.01	0.63 ± 0.01
		dt	reliefF	15	0.71 ± 0.02	0.63 ± 0.03
**radiomics from single modalities/clinical**	**ADC**	dt	no_alg	all	0.74 ± 0.02	0.67 ± 0.03
		**nb**	**reliefF**	**10**	**0.73 ± 0.02**	**0.69 ± 0.03**
		svm	reliefF	25	0.75 ± 0.02	0.67 ± 0.03
	**PC**	nb	no_alg	all	0.68 ± 0.02	0.54 ± 0.04
		mlp	gini_index	20	0.66 ± 0.01	0.56 ± 0.02
		dt	chi	5	0.66 ± 0.02	0.55 ± 0.04
	**SUB**	nb	no_alg	all	0.71 ± 0.03	0.58 ± 0.07
		nb	reliefF	5	0.64 ± 0.02	0.62 ± 0.02
		mlp	chi	30	0.69 ± 0.03	0.62 ± 0.04
	**T2**	nb	no_alg	all	0.68 ± 0.04	0.57 ± 0.05
		nb	fisher	50	0.68 ± 0.03	0.64 ± 0.02
		dt	reliefF	50	0.71 ± 0.02	0.63 ± 0.03
**radiomics/clinical**	**ALL**	nb	no_alg	all	0.7 ± 0.02	0.59 ± 0.04
		**mlp**	**gini_index**	**10**	**0.73 ± 0.02**	**0.68 ± 0.02**
		dt	reliefF	50	0.71 ± 0.02	0.64 ± 0.02
**radiomics**	**ALL**	dt	no_alg	all	0.66 ± 0.02	0.6 ± 0.02
		**rf**	**reliefF**	**30**	**0.7 ± 0.01**	**0.62 ± 0.02**
		knn	chi	5	0.64 ± 0.04	0.6 ± 0.04

Second feature selection step						
**Feature type**	**Image modality**	**Algorithm**	**Feature selection algorithm**	**Feature threshold t2**	**accuracy ± variance**	**f1score ± variance**
**radiomics from single modalities**	**ADC**	**knn**	**mi**	**27**	**0.74 ± 0.03**	**0.69 ± 0.05**
		svm	mi	42	0.73 ± 0.03	0.68 ± 0.04
**radiomics/clinical ***	**ALL**	dt	cfs	5	**0.8 ± 0.02**	0.75 ± 0.04

*only radiomics were selected

Predictor building with the Leave-One-Out approach						
**Feature type**	**Image modality**	**Algorithm**	**Feature selection algorithm**	**Feature number**	**accuracy**	**f1score**
**cliniche/radiomiche ***	**ALL**	**rf**	**no_alg**	**5**	**0,8**	**0,64**
		svm	no_alg	5	0,74	0,64

in **bold** = best result (F1score) for each “Feature type” section
in **red** = best result (F1score) among ALL the “Feature type” sections

Table S3.

Results of the first and second feature selection steps (first two sub-tables), and of the final classification (last sub-table) for the KI67+/KI67- classification task.

Positive/Negative KI67 marker. Number of patients=78 (P=14%,N=86%) First feature selection step						
**Feature type**	**Image modality**	**Algorithm**	**Feature selection algorithm**	**Feature threshold t1**	**accuracy ± variance**	**f1score ± variance**
**radiomics from single modalities**	**ADC**	svm	no_alg	all	0.78 ± 0.03	0.61 ± 0.06
		nb	chi	10	0.86 ± 0.01	0.69 ± 0.05
		rf	reliefF	45	0.82 ± 0.03	0.68 ± 0.06
	**PC**	svm	no_alg	all	0.9 ± 0.01	0.69 ± 0.06
		**svm**	**chi**	**10**	**0.89 ± 0.01**	**0.77 ± 0.06**
		rf	fisher	5	0.86 ± 0.02	0.77 ± 0.05
	**SUB**	dt	no_alg	all	0.82 ± 0.01	0.64 ± 0.05
		rf	fisher	30	0.88 ± 0	0.72 ± 0.03
		dt	reliefF	50	0.9 ± 0.01	0.7 ± 0.07
	**T2**	svm	no_alg	all	0.76 ± 0.03	0.57 ± 0.06
		nb	chi	25	0.81 ± 0.02	0.67 ± 0.06
		dt	reliefF	40	0.85 ± 0.02	0.66 ± 0.06
**radiomics from single modalities/clinical**	**ADC**	svm	no_alg	all	0.79 ± 0.03	0.64 ± 0.07
		dt	chi	20	0.81 ± 0.02	0.68 ± 0.04
		nb	reliefF	15	0.82 ± 0.02	0.67 ± 0.06
	**PC**	svm	no_alg	all	0.87 ± 0.01	0.67 ± 0.05
		**mlp**	**gini_index**	**5**	**0.85 ± 0.02**	**0.75 ± 0.05**
		nb	fisher	10	0.86 ± 0.02	0.74 ± 0.06
	**SUB**	rf	no_alg	all	0.82 ± 0.02	0.63 ± 0.07
		rf	fisher	30	0.85 ± 0.01	0.69 ± 0.03
		dt	fisher	5	0.81 ± 0.01	0.68 ± 0.04
	**T2**	dt	no_alg	all	0.79 ± 0	0.59 ± 0.03
		rf	chi	5	0.8 ± 0.02	0.71 ± 0.05
		dt	fisher	30	0.8 ± 0.02	0.67 ± 0.05
**radiomics/clinical**	**ALL**	dt	no_alg	all	0.82 ± 0.01	0.57 ± 0.03
		**dt**	**chi**	**50**	**0.85 ± 0.01**	**0.72 ± 0.03**
		dt	chi	35	0.86 ± 0.02	0.71 ± 0.07
**radiomics**	**ALL**	dt	no_alg	all	0.77 ± 0.02	0.53 ± 0.04
		**dt**	**fisher**	**20**	**0.87 ± 0.02**	**0.79 ± 0.05**
		dt	fisher	40	0.85 ± 0.01	0.7 ± 0.05

Second feature selection step						
**Feature type**	**Image modality**	**Algorithm**	**Feature selection algorithm**	**Feature threshold t2**	**accuracy ± variance**	**f1score ± variance**
**radiomics**	**ALL**	**svm**	**lr**	**21**	**0.84 ± 0.02**	**0.7 ± 0.06**
		mlp	cfs	**7**	0.82 ± 0.02	0.7 ± 0.06
**radiomics from single modalities**	**PC**	**mlp**	**cfs**	**1**	**0.87 ± 0.02**	**0.79 ± 0.05**
		mlp	cfs	2	0.86 ± 0.03	0.78 ± 0.06

Predictor building with the Leave-One-Out approach						
**Feature type**	**Image modality**	**Algorithm**	**Feature selection algorithm**	**Feature number**	**accuracy**	**f1score**
**radiomics from single modalities**	**PC**	knn/svm *	no_alg	1	0,88	0,93
		mlp	no_alg	1	0,82	0,89
		svm *	no_alg	2	0,88	0,93
		**mlp**	**no_alg**	**2**	**0,85**	**0,9**

* predicts only a single class
in **bold** = best result (F1score) for each "Feature type" section
in red = best result (F1score) among ALL the "Feature type" sections

Table S4.

Results of the first and second feature selection steps (first two sub-tables), and of the final classification (last sub-table) for the PR+/PR- classification task.

Positive/Negative PR marker. Number of patients=80 (P=50%,N=50%) First feature selection step						
**Feature type**	**Image modality**	**Algorithm**	**Feature selection algorithm**	**Feature threshold t1**	**accuracy ± variance**	**f1score ± variance**
**radiomics from single modalities**	**ADC**	svm	no_alg	all	0.6 ± 0.03	0.59 ± 0.03
		mlp	fisher	25	0.65 ± 0.02	0.64 ± 0.02
		svm	fisher	30	0.64 ± 0.03	0.62 ± 0.03
	**PC**	nb	no_alg	all	0.61 ± 0.04	0.59 ± 0.05
		**knn**	**fisher**	**5**	**0.74 ± 0.03**	**0.73 ± 0.03**
		knn	fisher	25	0.7 ± 0.03	0.67 ± 0.04
	**SUB**	mlp	no_alg	all	0.54 ± 0.05	0.53 ± 0.06
		knn	fisher	5	0.63 ± 0.04	0.61 ± 0.05
		svm	fisher	15	0.61 ± 0.04	0.6 ± 0.04
	**T2**	mlp	no_alg	all	0.56 ± 0.02	0.55 ± 0.02
		mlp	fisher	50	0.7 ± 0.02	0.68 ± 0.03
		mlp	gini_index	35	0.69 ± 0.03	0.67 ± 0.04
**radiomics from single modalities/clinical**	**ADC**	svm	no_alg	all	0.61 ± 0.04	0.6 ± 0.04
		mlp	fisher	25	0.65 ± 0.02	0.64 ± 0.02
		svm	fisher	30	0.64 ± 0.03	0.62 ± 0.03
	**PC**	nb	no_alg	all	0.61 ± 0.04	0.59 ± 0.05
		**knn**	**fisher**	**5**	**0.74 ± 0.03**	**0.73 ± 0.03**
		dt	chi	5	0.66 ± 0.01	0.65 ± 0.01
	**SUB**	mlp	no_alg	all	0.5 ± 0.05	0.49 ± 0.05
		knn	fisher	5	0.63 ± 0.04	0.61 ± 0.05
		mlp	reliefF	5	0.61 ± 0.03	0.6 ± 0.03
	**T2**	mlp	no_alg	all	0.56 ± 0.02	0.54 ± 0.02
		mlp	fisher	50	0.7 ± 0.02	0.68 ± 0.03
		mlp	gini_index	25	0.66 ± 0.03	0.65 ± 0.03
**radiomics/clinical**	**ALL**	mlp	no_alg	all	0.64 ± 0.02	0.62 ± 0.03
		**mlp**	**reliefF**	**15**	**0.69 ± 0.06**	**0.67 ± 0.07**
		mlp	fisher	50	0.64 ± 0.04	0.61 ± 0.05
**radiomics**	**ALL**	nb	no_alg	all	0.59 ± 0.02	0.53 ± 0.04
		**mlp**	**reliefF**	**15**	**0.69 ± 0.06**	**0.67 ± 0.07**
		mlp	fisher	50	0.64 ± 0.04	0.61 ± 0.05

Second feature selection step						
**Feature type**	**Image modality**	**Algorithm**	**Feature selection algorithm**	**Feature threshold t2**	**accuracy ± variance**	**f1score ± variance**
**radiomics from single modalities**	**PC**	**knn**	**lr**	**3**	**0.75 ± 0.02**	**0.74 ± 0.03**
		nb	cfs	6	0.73 ± 0.04	0.71 ± 0.04
**radiomics**	**ALL**	**rf**	**lr**	**9**	**0.75 ± 0.02**	**0.73 ± 0.02**

Predictor building with the Leave-One-Out approach						
**Feature type**	**Image modality**	**Algorithm**	**Feature selection algorithm**	**Feature number**	**accuracy**	**f1score**
**radiomics from single modalities**	**PC**	**mlp***	**no_alg**	**3**	**0,73**	**0,73**
		knn	no_alg	3	0,74	0,72
		**nb**	**no_alg**	**6**	**0,73**	**0,74**
		rf	no_alg	6	0,68	0,68

*the method with the lowest feature number was selected as the best
in **bold** = best result (F1score) for each "Feature type" section
in red = best result (F1score) among ALL the "Feature type" sections

Table S5. Retrospective studies on predicting BC molecular subtypes. MORPH = morphology, IS = intensity-based statistics, LoG = Laplacian of Gaussian, WL = wavelet, ICCs = intra and inter correlations, PCCs = Pearson correlation coefficients, ANOVA = analysis of variance, mRMR = maximum relevance–minimum redundancy.

First Author Year	*# Patients*	*MRI Modality*	*Magnetic Field*	*Radiomic Feature Family*	*Feature Selection Algorithm*	*Validation on external dataset*	*Aim*	*Outcomes*
Huang et al[48] (2021)	162	DCE T1, T2w fat sat, ADC maps	3 T	MORPH, IS, GLCM, GLSZM, GLRLM, NGTDM, NGLDM, LoG and WL	ICCs, LASSO-RFE, mRMR, Boruta method and PCCs.	No	To predict molecular subtypes (ER, PR, HER2) and androgen receptor (AR) expression.	The MLP classifiers showed the best diagnostic performance in three molecular subtype classification tasks and in discriminating AR expression (AUC =0.907; accuracy = 85.8%).
Kayadibi et al[50] (2022)	154	DCE T1w fat sat FLASH, ADC maps	1.5 T	MORPH, IS, GLCM, GLSZM, GLRLM, NGTDM, NGLDM, LoG and WL	ICC, LASSO	No	To investigate the value of MRI-based radioproteomics in predicting Ki67 expression levels.	The best model performance was achieved with 15 selected features from the ADC sequence based on the Ki67 cut-off value of 14%.
Fan et al[51] (2019)	144	DCE T1w fat sat, ADC maps	3 T	MORPH, IS, GLCM	Multi task feature selection	No	To improve prediction accuracy of histological grade and Ki67 status based on tumor radiomic analysis.	Multi task learning models improve the prediction of Ki67 status and tumor grade.
Zhong et al[52] (2022)	443	DCE-MRI,	3 T	IS, GLCM, Gabor and Law	Two-sample t-test, LASSO, Stepwise	No	To establish and validate the multiregional radiomic signatures for the preoperative identification of the ER and PR status.	Multiregional radiomic signatures achieved the best AUCs for evaluating the ER (0.851 and 0.833) and PR (0.848 and 0.763) status.
Park et al[54] (2018)	294	DCE T1w, T2w, PC T1w, SUB DCE T1w	1.5 T	MORPH, IH, GLCM, GLSZM	ICC value of Rad-score	No	To establish a radiomics nomogram that incorporates the radiomics signature for disease free survival (DFS), MRI and	Higher Rad-scores were significantly associated with worse DFS in both the training and validation sets (P=0.002 and 0.036, respectively). The radiomics nomogram estimated DFS

							clinicopathological findings.	better than the clinicopathological or Rad-score–only nomograms.
Xie et al[55] *(2019)*	134	T2w, T1w, DCE T1w, DWI	3 T	MORPH, IS and texture features	ANOVA, CV, t-test, LASSO and ridge regression and elastic net, PCC	No	To classify immunohistochemical subtypes with ML analysis of mpMRI radiomics.	For 4-IHC classification task, the best accuracy of 72.4% was achieved based on linear discriminant analysis. For comparing TN to non-TN cancers, the highest accuracy of 91.0% was obtained with SVM and medium KNN.
Liu et al[56] *(2019)*	586	DCE T1w, T2w, DWI		MORPH, IH, texture, Gabor and Law	Univariate analysis with the Mann-Whitney U test, PCC, Boruta method	Yes	To evaluate radiomics of mpMRI for pretreatment prediction of pathologic complete response to neoadjuvant chemotherapy.	Radiomic signature combining multiparametric MRI achieved an AUC of 0.79.
Ni et al[57] *(2020)*	112	DWI, T2w fat sat, T1w, T1w pre contrast fat sat, DCE T1w fat sat	3 T	MORPH, IH, texture, GLSZM, GLCM and RLM	Remove outlier, PCC, mean center and standard deviation scale and noise processing	No	To investigate the feasibility of using a Fisher discriminant analysis model based on radiomic features of DWI for predicting the clinicopathological subtypes.	The overall accuracy for predicting the clinicopathological subtypes was 96.4% by Fisher discriminant analysis, and the weighted accuracy was 96.6%.
Saha et al[58] *(2018)*	922	T1w, T1w fat sat pre contrast, T1w post contrast, DCE	1.5 – 3 T	MORPH, IS, texture (using spatial and no-spatial information)	--	Yes	To investigate the associations of MRI-based imaging phenotypes with molecular, genomic, and proliferation markers (ER, PR, HER2 and Ki67).	Luminal A subtype with AUC = 0.697 (95% CI: 0.647–0.746, p < .0001), triple negative breast cancer with AUC = 0.654 (95% CI: 0.589–0.727, p < .0001), ER status with AUC = 0.649 (95% CI: 0.591–0.705, p < .001), and PR status with AUC = 0.622 (95% CI: 0.569–0.674, p < .0001).
